# Socioeconomic disparities in treatment and survival of oesophageal and gastric cancer in the Netherlands: a nationwide population-based study

**DOI:** 10.1016/j.lanepe.2026.101662

**Published:** 2026-03-26

**Authors:** Dillen C. van der Aa, Mats D. Teeken, Hanneke W.M. van Laarhoven, Suzanne S. Gisbertz, Sjoerd M. Lagarde, Nadia Haj Mohammad, Peter S.N. van Rossum, Rob H.A. Verhoeven, Mark I. van Berge Henegouwen

**Affiliations:** a Department of Surgery, Amsterdam UMC, Location University of Amsterdam, Meibergdreef 9, Amsterdam, the Netherlands; b Cancer Centre Amsterdam, Cancer Treatment and Quality of Life, Amsterdam, the Netherlands; c Department of Gastroenterology and Hepatology, Amsterdam Gastroenterology Endocrinology Metabolism, Amsterdam UMC, Location University of Amsterdam, Meibergdreef 9, Amsterdam, the Netherlands; d Department of Medical Oncology, Amsterdam UMC, Location University of Amsterdam, Amsterdam, the Netherlands; e Department of Surgery, Division of Surgical Oncology and Gastrointestinal Surgery, Erasmus Medical Centre Cancer Institute, Rotterdam, the Netherlands; f Department of Medical Oncology, University Medical Centre Utrecht, Utrecht University, Utrecht, the Netherlands; g Department of Radiation Oncology, Amsterdam UMC Location Vrije Universiteit Amsterdam, the Netherlands; h Department of Research and Development, Netherlands Comprehensive Cancer Organization (IKNL), Utrecht, the Netherlands

**Keywords:** Socioeconomic status, Oesophageal cancer, Gastric cancer, Treatment, Outcomes

## Abstract

**Background:**

In the Netherlands, all inhabitants are required to have health insurance that covers standard medical care. While this regulated system aims to ensure equal access, recent evidence suggests that socioeconomic status (SES) disparities still exist. This study investigates SES-related differences in treatment and survival among patients with gastric or oesophageal cancer.

**Methods:**

This nationwide population-based study included gastroesophageal cancer patients diagnosed in 2015–2022. Patients were stratified into three SES-groups using median disposable household income per postal code area, an additional stratification was performed by histological subtype. Multivariable logistic regression was used to assess the likelihood of receiving specific treatments for different tumour types. Age-standardized relative survival was calculated as the ratio of observed survival to expected survival based on age, sex, calendar year, and median household income group in the general population.

**Findings:**

In total, 30,184 gastroesophageal cancer patients were categorized. In curable gastric cancer, patients with middle income had a significantly higher likelihood of receiving surgery compared to low income (OR 1.48 (95% CI 1.21–1.80), p < 0.001). For oesophageal cancer, both middle- (OR 1.17 (95% CI 1.05–1.30), p < 0.001) and high-income patients (OR 1.27 (95% CI 1.13–1.43), p < 0.001) were more likely to undergo surgery than those with low income. In palliative settings, middle- and high-income patients were also more likely to receive systemic therapy, in oesophageal cancer (OR 1.38 (95% CI 1.21–1.57), p < 0.001; OR 1.53 (95% CI 1.34–1.74), p < 0.001) and in gastric for high income (OR 1.27 (95% CI 1.05–1.55), p = 0.014). Relative survival analyses further demonstrated that middle- and high-income groups consistently had higher median, and 5-year survival compared to the low-income group.

**Interpretation:**

Despite universal health insurance, socioeconomic disparities in treatment and survival of gastroesophageal cancer patients persist in the Netherlands, reflecting not inequitable access but broader life-course health disparities that influence treatment fitness and outcomes. These findings highlight the need to address broader social and systemic determinants to improve equity in cancer care, in the Netherlands and globally.

**Funding:**

This study received no funding.

**Research in context**

**Evidence before this study**

We searched PubMed, MEDLINE, and Embase (English language; last search Feb 1, 2025) using combinations of “socioeconomic status/income”, “inequalities/disparities”, “esophageal/oesophageal cancer”, ‘’gastrointestinal cancer’’ and “gastric/stomach cancer”. Prior studies, predominantly from the USA and several European or Asian cohorts, consistently reported that lower socioeconomic status (SES) is linked to later stage at diagnosis, lower use of guideline-concordant or definitive treatment, and worse survival in upper-GI cancers. In the Netherlands, earlier work suggested SES-related differences but was limited by regional samples, older eras, and insufficient separation of curative versus palliative pathways and histological subtypes. Contemporary nationwide estimates within a universal, mandatory insurance system were lacking.

**Added value of this study**

Using nationwide Netherlands Cancer Registry data (2015–2022), we analysed 30.184 patients with gastric or oesophageal cancer, stratified by income-based SES, histology, and treatment intent. Compared with low-income patients, middle- and high-income groups were more likely to receive surgery in potentially curable disease and systemic therapy in palliative disease. These treatment differences coincided with consistently lower age-standardised relative survival in the low-income group. Notably, disparities persisted after adjustment for comorbidity and performance status, indicating that measurable clinical factors do not fully account for inequities within a universal insurance framework. The study provides contemporary, population-level estimates using Ederer-2 relative survival and explicitly separates curative from palliative care pathways.

**Implications of all the available evidence**

Even in a universal, mandatory insurance system, substantial SES-related inequities in treatment allocation and survival for gastroesophageal cancer persist. These findings suggest that financial coverage alone does not eliminate outcome differences. Addressing disparities will likely require attention to structural and pathway-level factors, including timely staging, uniform referral to specialist centres, and optimisation of comorbidities in vulnerable populations. Improved routine capture of individual-level socioeconomic indicators, such as educational attainment and health literacy, alongside area-level income would strengthen monitoring and facilitate development of more precisely targeted equity-focused interventions.

## Introduction

Gastric and oesophageal cancers have 5-year survival rates of 23% and 24% in the Netherlands.^1^ Curative-intent treatment generally involves multimodal therapy, combining surgery with (neo)adjuvant chemo (radio)therapy, as recommended by clinical guidelines.^2^, ^3^, ^4^, ^5^, ^6^, ^7^, ^8^, ^9^ For many patients, however, the disease is already advanced at diagnosis, limiting eligibility for curative options. In these cases, palliative therapies, including chemotherapy, immunotherapy, or radiotherapy, are used to relieve symptoms and potentially extend survival.

The role of social determinants of health, such as socioeconomic status (SES), in access to cancer care is increasingly recognized.^10^ A recent trilogy of reports from the Netherlands Cancer Registry (NCR) highlights unfavourable outcomes for individuals with lower SES regarding cancer incidence, diagnosis, treatment, and survival.^11^ While these reports offer a broad overview of SES-related disparities in cancer care, they do not specifically address gastric and oesophageal cancers. These cancers are particularly relevant given their poor prognosis, complex multimodal treatment, and frequent presentation at an advanced stage, making disparities in treatment delivery especially impactful on outcomes.^1^ Studies from the United States indicate that individuals with lower SES have a higher risk of developing gastric and oesophageal cancer, are more frequently diagnosed at a later stage, and are more often excluded from receiving treatment.^12^, ^13^, ^14^

Although all inhabitants of the Netherlands are covered by the same mandatory health insurance, effectively reducing financial barriers to care,^15^ earlier studies suggest that gastric and oesophageal cancer patients with lower SES have a reduced chance of undergoing a curative intent resection and have a worse survival.^16^, ^17^, ^18^ However, these studies are limited to regional cohorts, focus only on surgery or chemotherapy regimens, lack distinction between curative and palliative patients, and rely mostly on older data (2007 for gastric cancer and 2010 for oesophageal cancer). Since then, cancer care has evolved with centralized surgical treatment, new treatment methods have been developed and are available in the Netherlands.^19^, ^20^, ^21^, ^22^ Understanding the socioeconomic disparities in a system with minimal financial barriers underscores the importance of non-financial factors driving poor outcomes for lower SES globally and may provide valuable insights to health care policy makers aiming to improve equity in cancer care.

This study aims to assess SES-related disparities in gastric and oesophageal cancer care delivery in the Netherlands. We hypothesize that lower SES is associated with reduced chance of receiving surgery in curative patients and receiving systemic therapy in palliative patients, and poorer survival in both patient groups.

## Methods

### Patient selection

**Fig. 1:**
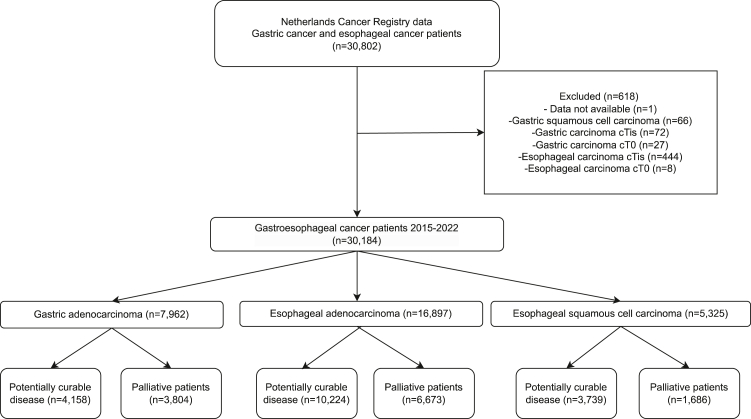
Flowchart of data distribution and patients. Abbreviations: cT, clinical Tumor stage.

This nationwide, population-based study analysed treatment disparities in patients with gastric adenocarcinoma or oesophageal squamous cell carcinoma or adenocarcinoma stratified by SES. The NCR provides accurate data on clinical features of newly diagnosed gastric and oesophageal cancer patients in the Netherlands. Our database included all patients with gastric and/or oesophageal cancer diagnosed in 2015–2022. Exclusion criteria were clinical T0 and Tis stage, and gastric squamous cell carcinoma. Inclusion and exclusion of patients is shown in the flowchart (Fig. 1).

New cancer cases were identified through the National Automated Pathology Archive, which provides weekly notifications of all newly diagnosed cases based on pathologic confirmation. Additionally, patients diagnosed through other methods than pathology, were identified via the Dutch National Hospital Care Registry. More detailed clinical and tumour-related information was retrieved from medical records by certified data managers of the NCR. Patient survival status was updated annually using data from the Dutch Personal Records Database, with survival follow-up available until February 1, 2025, at the time of data extraction.

The Privacy Review Board of the NCR and the Scientific Committee of the Dutch Upper GI Cancer Group approved this study. No ethical approval was required under Dutch law. Individual patient consent was not required. All study procedures complied with the ethical guidelines established by the relevant institutional review board, as well as the principles outlined in the Declaration of Helsinki (1964) and its subsequent revisions.

Patients were included in the potentially curative group based on the absence of cT4b or metastatic disease. Stratification was based on type of cancer and the curability of the disease. Within oesophageal cancer patients, an extra stratification was done based on histology type (i.e., adenocarcinoma versus squamous cell carcinoma). Treatment intent was based on therapeutic pathways recorded in the NCR. As the NCR does not specify whether chemoradiotherapy without surgery was intended as definitive or neoadjuvant, minor misclassification may have occurred. This is unlikely to differ systematically across SES groups.

### Socioeconomic status

For income classification, standardized income data from Statistics Netherlands (CBS) for the year 2019 was used. This refers to the disposable (net) annual household income per postal code area, adjusted for household size and composition. CBS determined the median household income for each postal code area on average 25 households. Because the number of households per postal code area is often limited, CBS incorporates the 99% confidence interval of the median income into the classification. These income levels were then categorized into quintiles (five equal groups). If the 99% confidence interval of a postal code area crossed a quintile threshold, an overlapping category was created. As a result, income categories may partially overlap. Using this method, the CBS initially defined twelve income categories based on the national income distribution. Income categories were classified according to national income percentiles. The low to lower-middle income group (0th–40th percentile) comprised individuals with an estimated median income of less than €24,300. The middle-income group (40th–60th percentile) included those with an estimated median income ranging from €24,300 to €31,000. When the median income was based on fewer than ten households, income was categorized as “unclassifiable.” The upper-middle to high-income group (60th–100th percentile) represented individuals with an estimated median income of greater than €31,000. However, to simplify analysis and eliminate overlap in income ranges, these categories were reduced to three income groups: low, middle, and high. To establish the three groups, the first two quintiles of the CBS definition were combined, as well as the last two quintiles. This reduction was performed to avoid overlapping income boundaries and to ensure statistical robustness and interpretability, particularly in multivariable and stratified analyses.

### Statistical analysis

For potentially curable gastric cancer, the main outcome was the proportion of patients receiving (neo)adjuvant therapy with a resection. For potentially curable oesophageal cancer, the main outcome of this study was the proportion of patients receiving neoadjuvant chemoradiotherapy and resection, ±nivolumab or only chemoradiotherapy for SANO-trial patients.^23^ The proportion of patients receiving systemic therapy was the main outcome for palliative patients. The proportion of patients receiving diverse types of treatment and best supportive care was calculated within each SES group. Baseline characteristics and treatment utilization of different treatment modalities were summarized with descriptive statistics, means (SD) for normally distributed variables, medians (range) for non-normally distributed variables, and counts (percentages) for categorical data. Group comparisons used independent t-tests or Mann–Whitney U tests for continuous variables, and chi-square or Fisher's exact tests for categorical variables. The odds-ratio (OR) of receiving treatment was calculated using univariable and multivariable logistic regression models, adjusting for both demographic and clinical covariates (i.e., age, sex, Charlson comorbidity index (CCI), performance status, histology type Lauren classification, tumour location, and stage) as they may be associated with determining treatment utilization and thus potentially act as confounders. To explore effect modification, interaction terms (e.g., SES × gender) were included in regression models to assess whether the association between SES and treatment varied across subgroups. Effect modification by calendar time was evaluated using an interaction term between SES and year of diagnosis. Although this is not a multivariable prediction model study, key statistical methods were transparently reported in accordance with the principles of clear and reproducible research, as recommended by the TRIPOD statement.^24^ Potential confounders (age (continuous & categorical (potentially curable)), sex, WHO performance status, number of comorbidity categories, therapy region and tumour, clinical Tumour stage, clinical Nodal stage, histology and differentiation) were adjusted for using a multivariable Cox proportional hazards model. Age-standardized relative survival was calculated as the ratio of the survival observed among the patients with gastroesophageal cancer to the survival that would have been expected based on age, gender, year and median household income group of the corresponding general population (Ederer 2 method).^25^ Missing values were rare (<2%). Cases with missing data were retained in descriptive analyses but excluded from multivariable models using listwise deletion. Given the large sample size and the low proportion of missing data, imputation was not performed. The data were examined using IBM SPSS Statistics, version 28.0, relative survival analyses were performed with Stata, version 17. Statistical significance was defined as p < 0.050.

### Role of the funding source

This study received no funding.

## Results

### Baseline characteristics

This study included 30,184 esophagogastric patients categorized by SES. Among 7962 gastric cancer patients, 3090 (38.8%) were categorized as low SES, 2803 (35.2%) as middle SES, and 2069 (26.0%) as high SES. The proportion of male patients varied by income group, with fewer males in the low-income group (57.1%) compared to the middle-income (61.1%) and high-income groups (60.7%) (p = 0.003). The median age was lower in the higher income-group, from 74 years in the low-income group to 72 years in the high-income group (p < 0.001). A higher proportion of low-income patients had ≥2 comorbidities (26.8%) compared to middle-income (22.0%) and high-income (17.9%) patients (p < 0.001). Additionally, low-income patients had worse WHO performance status, with only 18.5% scoring 0 compared to 27.0% and 26.8% in the middle- and high-income groups, respectively (p < 0.001) (Table 1). Patients of the low-income group were less often staged with cM1 (43.6%) compared to the high-income group (47.2%, p = 0.039) (Table 1).

**Table 1:** Baseline characteristics of included patients (n = 30,184).

	Gastric cancer (n = 7962)			p-value	Esophageal cancer (n = 22,222)			p-value
	Low (n = 3090) n (%)	Middle (n = 2803) n (%)	High (n = 2069) n (%)		Low (n = 7036) n (%)	Middle (n = 8183) n (%)	High (n = 7003) n (%)	
Characteristics								
Gender				0.003				<0.001
Male	1765 (57.1)	1713 (61.1)	1256 (60.7)		4949 (70.3)	6165 (75.3)	5330 (76.1)	
Female	1325 (42.9)	1090 (38.9)	813 (39.3)		2087 (29.7)	2018 (24.7)	1673 (23.9)	
Age (med [range])	74 [19–100]	73 [21–96]	72 [20–96]	<0.001	71 [21–101]	70 [24–102]	69 [18–98]	<0.001
Number of CCI categories				<0.001				<0.001
0	1146 (38.8)	1245 (46.0)	1018 (52.1)		2515 (35.7)	3525 (43.1)	3382 (48.3)	
1	1015 (34.4)	865 (32.0)	586 (30.0)		2321 (33.0)	2637 (32.2)	2071 (29.6)	
≥2	793 (26.8)	594 (22.0)	350 (17.9)		1974 (28.1)	1757 (21.5)	1204 (17.2)	
Unknown	136 (4.4)	99 (3.5)	115 (5.6)		226 (3.2)	264 (3.2)	346 (4.9)	
WHO performance status				<0.001				<0.001
0	571 (18.5)	756 (27.0)	555 (26.8)		1618 (23.0)	2511 (30.7)	2411 (34.4)	
1	772 (25.0)	738 (26.3)	531 (25.7)		2109 (30.0)	2562 (31.3)	2123 (30.3)	
≥2	603 (19.5)	425 (15.1)	294 (14.2)		1452 (20.6)	1226 (15.0)	870 (12.4)	
Unknown	1144 (37.0)	885 (31.6)	689 (33.3)		1857 (26.4)	1884 (23.0)	1599 (22.8)	
Histology								<0.001
Adenocarcinoma	–	–	–		5175 (73.6)	6279 (76.7)	5443 (77.7)	
Squamous cell carcinoma	–	–	–		1861 (26.4)	1904 (23.3)	1560 (22.3)	
Lauren classification (adenocarcinoma)				0.041				0.104
Intestinal	1160 (37.7)	1061 (37.9)	752 (36.4)		2669 (37.9)	3392 (41.5)	2815 (40.2)	
Diffuse	1288 (41.8)	1216 (43.5)	944 (45.7)		750 (10.7)	915 (11.2)	816 (11.7)	
Mixed	122 (4.0)	115 (4.1)	76 (3.7)		139 (2.0)	166 (2.0)	166 (2.4)	
Unknown	509 (16.5)	404 (14.4)	293 (14.2)		1615 (22.9)	1700 (22.0)	1640 (23.4)	
cT stage				0.458				<0.001
T1	86 (2.8)	74 (2.6)	56 (2.7)		287 (4.1)	272 (3.3)	233 (3.3)	
T2	613 (19.8)	580 (20.7)	437 (21.2)		1479 (21.0)	1718 (21.0)	1458 (20.8)	
T3	1070 (34.6)	1024 (36.5)	762 (36.8)		3525 (50.1)	4364 (53.3)	3813 (54.4)	
T4	449 (14.5)	381 (13.6)	282 (13.6)		448 (6.4)	458 (5.6)	478 (6.8)	
T4a	–	–	–		180 (2.6)	199 (2.4)	184 (2.6)	
T4b	–	–	–		268 (3.8)	259 (3.2)	294 (4.2)	
Tx	872 (28.2)	744 (26.5)	532 (25.7)		1296 (18.4)	1369 (16.7)	1020 (14.6)	
cN stage				0.046				<0.001
N0	1403 (45.4)	1311 (46.8)	970 (46.9)		2311 (32.8)	2471 (30.2)	2189 (31.3)	
N1	652 (21.1)	641 (22.9)	454 (21.9)		2087 (29.7)	2548 (31.1)	2172 (31.0)	
N2	517 (16.7)	472 (16.8)	340 (16.4)		1636 (23.3)	2030 (24.8)	1717 (24.5)	
N3	107 (3.5)	92 (3.3)	74 (3.6)		398 (5.7)	558 (6.8)	517 (7.4)	
Nx	411 (13.3)	287 (10.2)	231 (11.2)		604 (8.6)	576 (7.0)	925 (13.2)	
cM stage				0.039				<0.001
M0	1742 (56.4)	1544 (55.1)	1092 (52.8)		4698 (66.8)	5200 (63.5)	4405 (62.9)	
M1	1348 (43.6)	1259 (44.9)	977 (47.2)		2338 (33.2)	2983 (36.5)	2598 (37.1)	
Curable disease				0.281				<0.001
Potentially curable	1644 (53.2)	1478 (52.7)	1038 (50.2)		4548 (64.6)	5071 (62.0)	4244 (60.6)	
Palliative	1446 (46.8)	1327 (47.3)	1031 (49.8)		2488 (35.4)	3112 (38.0)	2759 (39.4)	

Abbreviations: CCI, comorbidity categories index; WHO, World Health Organization.

p-values were calculated using a 2-sided Chi^2^ test.

Among 22,222 oesophageal cancer patients, 7036 (31.7%) were categorized as low SES, 8183 (36.8%) as middle SES and 7003 (31.5%) as high SES. Male patients were more prevalent in higher-income groups (70.3% among low-income versus 76.1% among high-income, p < 0.001). Median age was also lower in the higher income groups (71 in low versus 69 years in the high-income, p < 0.001). Comorbidity burden was highest in the low-income group, with 28.1% having ≥2 comorbidities, compared to 17.2% in the high-income group (p < 0.001). A better WHO performance status was more common in higher-income groups, with 34.4% of high-income patients scoring 0, compared to 23.0% in the low-income group (p < 0.001). Histologic subtype also differed significantly, with adenocarcinoma more common in higher-income patients (77.7% compared to 73.6% in the low-income group; p < 0.001; Table 1). The distribution of clinical M stage differed significantly by SES (p = 0.039). Patients of the low-income group were less often staged with cM1 (33.2%) compared to the high-income group (37.1%, p < 0.001) (Table 1).

### Gastric cancer treatment and survival related to SES

In 4158 patients with potentially curable gastric cancer, (neo)adjuvant treatment with surgery was administered in 33.2% of low-income patients, 42.8% of middle-income patients, and 45.2% of high-income patients (p < 0.001). Best supportive care was more common in the low-income group (27.6%) compared to the middle-income (18.7%) and high-income group (20.2%) (p < 0.001) (Table 2). Multivariable logistic regression showed increased odds-ratio for undergoing a surgical resection for medium versus low-income (OR 1.48; 95% CI: 1.21–1.80, p < 0.001), but no statistically significant difference between high and low-income groups (OR 1.02; 95% CI: 0.82–1.27, p = 0.854; Table 3).

**Table 2:** Treatment strategies in potentially curable gastroesophageal cancer patients.

	Gastric adenocarcinoma (n = 4158)				Esophageal adenocarcinoma (n = 10,224)				Esophageal squamous cell carcinoma (n = 3739)			
	Low (n = 1644) n (%)	Middle (n = 1476) n (%)	High (n = 1038) n (%)	p-value	Low (n = 3238) n (%)	Middle (n = 3773) n (%)	High (n = 3213) n (%)	p-value	Low (n = 1310) n (%)	Middle (n = 1398) n (%)	High (n = 1031) n (%)	p-value
Treatment of choice				<0.001				<0.001				<0.001
Neoadjuvant chemoradiation and resection ± nivolumab	28 (1.7)	33 (2.2)	28 (2.7)		974 (30.1)	1505 (39.9)	1397 (43.5)		226 (17.3)	310 (22.2)	272 (26.4)	
(Neo)adjuvant chemotherapy and resection	517 (31.4)	600 (40.7)	441 (42.5)		187 (5.8)	239 (6.3)	283 (8.8)		6 (0.5)	5 (0.4)	11 (1.1)	
Surgery alone	414 (25.2)	395 (26.8)	254 (24.5)		98 (3.0)	103 (2.7)	109 (3.4)		32 (2.4)	27 (2.1)	27 (2.6)	
Endoscopic resection	56 (3.4)	55 (3.7)	28 (2.7)		289 (8.9)	325 (8.6)	257 (8.0)		38 (2.9)	42 (3.2)	29 (2.8)	
Definitive chemoradiation (>41.4 Gy)	–	–	–		341 (10.5)	325 (8.6)	260 (8.1)		348 (26.6)	406 (31.3)	284 (27.5)	
Chemoradiation without resection (41.4 Gy)	–	–	–		288 (8.9)	400 (10.6)	286 (8.9)		89 (6.8)	107 (8.2)	98 (9.5)	
Chemoradiation without resection (<41.4 Gy)	–	–	–		26 (0.8)	40 (1.1)	55 (1.7)		28 (2.1)	28 (2.2)	24 (2.3)	
Systemic therapy	81 (4.9)	62 (4.2)	65 (6.3)		71 (2.2)	84 (2.2)	63 (2.0)		20 (1.5)	19 (1.5)	17 (1.6)	
Best supportive care	548 (33.3)	331 (22.4)	250 (24.1)		964 (29.8)	752 (19.9)	503 (15.7)		523 (39.9)	354 (27.3)	269 (26.1)	

Abbreviations: +/−, with or without; Gy, gray.

p-values were calculated using a 2-sided Chi^2^ test.

**Table 3:** Multivariable logistic regression on chance of undergoing surgical resection in potentially curable gastroesophageal cancer patients.

	Gastric cancer		Esophageal cancer	
	Multivariable^a^		Multivariable^a^	
	OR (95% CI)	p-value	OR (95% CI)	p-value
Socioeconomic status				
Low-income	ref		ref	
Middle-income	1.48 (1.21–1.80)	<0.001	1.17 (1.05–1.30)	<0.001
High-income	1.02 (0.82–1.27)	0.854	1.27 (1.13–1.43)	<0.001

Abbreviations: CI, Confidence Interval; OR, Odds Ratio.

Interaction term between socioeconomic status and; year of diagnosis; gender; number of comorbidity categories; or World Health Organization score were not statistically significant and thus not included in the final adjusted model.

a In multivariable analysis corrected for: socioeconomic status, gender, age, clinical tumor stage, clinical nodal stage, tumor histology, Lauren classification, therapy regions, World Health Organization score and number of comorbidity categories.

For 3804 palliative gastric cancer patients, systemic therapy was administered in 32.8% of low-income patients, 39.9% of middle-income patients, and 42.1% of high-income patients (p < 0.001). Best supportive care was more frequently chosen in the low-income group (55.9%) compared to the middle-income (46.9%) and high-income groups (45.3%; p < 0.001; Table 4).

**Table 4:** Treatment strategies in palliative gastroesophageal cancer patients.

	Gastric adenocarcinoma (n = 3804)			p-value	Esophageal adenocarcinoma (n = 6673)			p-value	Esophageal squamous cell carcinoma (n = 1686)			p-value
	Low (n = 1446) n (%)	Middle (n = 1327) n (%)	High (n = 1031) n (%)		Low (n = 1937) n (%)	Middle (n = 2506) n (%)	High (n = 2230) n (%)		Low (n = 551) n (%)	Middle (n = 606) n (%)	High (n = 529) n (%)	
Treatment of choice				<0.001				<0.001				<0.001
Resection ± perioperative treatment	42 (2.9)	41 (3.0)	42 (4.1)		28 (1.4)	50 (1.9)	39 (1.7)		14 (2.5)	9 (14.9)	34 (6.4)	
Chemoradiation without resection					60 (3.1)	77 (3.1)	75 (3.4)		70 (12.7)	103 (17.0)	123 (23.3)	
Systemic therapy	474 (32.8)	530 (39.9)	434 (42.1)		682 (35.2)	1137 (45.4)	1148 (51.5)		84 (15.2)	127 (21.0)	108 (20.4)	
Best supportive care	898 (62.1)	717 (54.0)	523 (50.7)		1167 (60.3)	1242 (49.5)	958 (43.0)		383 (69.5)	367 (60.6)	264 (49.9)	

p-values were calculated using a 2-sided Chi^2^ test.

High-income patients had a higher odds of being treated with systemic therapy compared to low-income patients (OR 1.27; p = 0.014; Table 5).

**Table 5:** Multivariable logistic regression for being treated with systemic therapy in palliative gastroesophageal cancer patients.

	Gastric cancer		Esophageal cancer	
	Multivariable^a^		Multivariable^a^	
	OR (95% CI)	p-value	OR (95% CI)	p-value
Socioeconomic status				
Low-income	ref		ref	
Middle income	1.20 (0.99–1.44)	0.050	1.38 (1.21–1.57)	<0.001
High-income	1.27 (1.05–1.55)	0.014	1.53 (1.34–1.74)	<0.001

Abbreviations: CI, Confidence Interval; OR, Odds Ratio.

Interaction term between socioeconomic status; gender, number of comorbidity categories, and World Health Organization score were not statistically significant and thus not included in the final adjusted model.

a In multivariable analysis corrected for: socioeconomic status, gender, age, tumor histology, Lauren classification, therapy regions, World Health Organization score and number of comorbidity categories.

### Relative survival among gastric cancer patients

**Fig. 2:**
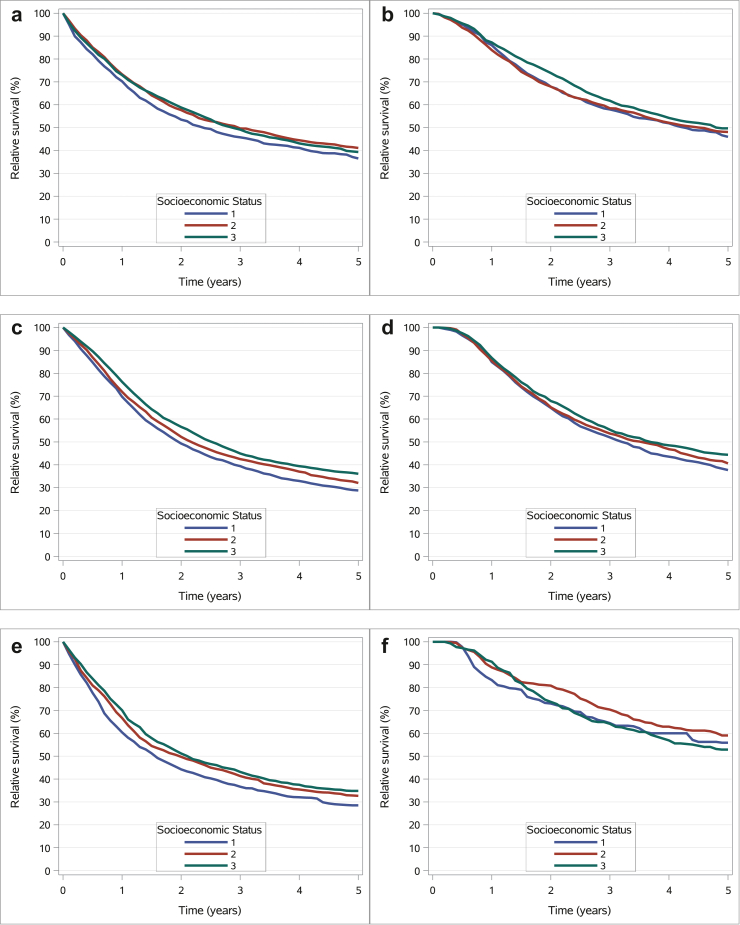
Relative survival curves in potentially curable gastroesophageal cancer patients. a. Relative survival of potentially curable GAC patients. b. Relative survival of potentially curable GAC patients who underwent surgery. c. Relative survival of potentially curable EAC patients. d. Relative survival of potentially curable EAC patients who underwent surgery. e. Relative survival of potentially curable ESCC patients. f. Relative survival of potentially curable ESCC patients who underwent surgery. Abbreviations: ESCC, esophageal squamous cell carcinoma; EAC, esophageal adenocarcinoma, GAC, gastric adenocarcinoma; socioeconomic status; 1 = low-income; 2 = middle-income; 3 = high-income.

Among patients with potentially curable gastric cancer, median relative survival was 28.5 months (95% CI: 24.4–32.4) in the low-income group, 35.6 months (95% CI: 32.4–38.8) in the middle-income group (p < 0.001 versus low), and 34.4 months (95% CI: 28.5–40.1) in the high-income group (p < 0.001 versus low). The 5-year relative survival rate was 36.5% (95% CI: 33.6–39.4) in the low-income group, compared to 41.1% (95% CI: 38.2–44.0) and 39.3% (95% CI: 35.8–42.8) in the middle- and high-income groups, respectively (p < 0.001 versus low) (Fig. 2 & Supplementary Table S6).

In the subgroup of patients who underwent surgery, median relative survival was 51.1 months (95% CI: 43.2–59.0) in the low-income group, 54.2 months (95% CI: 37.6–70.8) in the middle-income group (p = 0.34 versus low), and 57.6 months (95% CI: 42.3–73.1) in the high-income group (p = 0.01 versus low). Corresponding 5-year survival rates were 46.0% (95% CI: 42.4–49.4), 48.1% (95% CI: 44.7–51.4), and 49.7% (95% CI: 45.5–53.8), respectively (p = 0.01 versus low) (Fig. 2 & Supplementary Table S6).

**Fig. 3:**
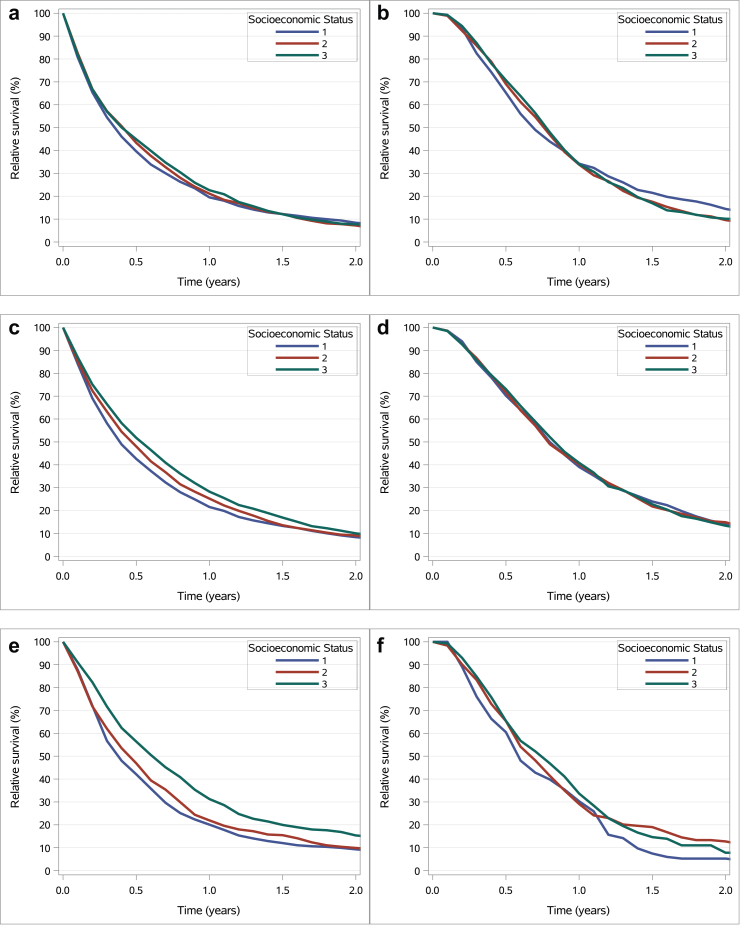
Relative survival curves in palliative gastroesophageal cancer patients. a. Relative survival of all metastatic GAC patients. b. Relative survival of all metastatic GAC patients who received systemic therapy. c. Relative survival of all metastatic EAC patients. d. Relative survival of all metastatic EAC patients who received systemic therapy. e. Relative survival of all metastatic ESCC patients. f. Relative survival of all metastatic ESCC patients who received systemic therapy. Abbreviations: ESCC, esophageal squamous cell carcinoma; EAC, esophageal adenocarcinoma, GAC, gastric adenocarcinoma; socioeconomic status; 1 = low-income; 2 = middle-income; 3 = high-income.

For patients with metastatic disease, median relative survival was 4.2 months (95% CI: 3.9–4.6) in the low-income group, 4.9 months (95% CI: 4.4–5.3) in the middle-income group (p = 0.11 versus low), and 4.8 months (95% CI: 4.1–5.5) in the high-income group (p = 0.051 versus low). The 1-year relative survival rate was 19.5% (95% CI: 17.4–21.7), 21.1% (95% CI: 18.9–23.5), and 22.6% (95% CI: 20.0–25.4), respectively (p = 0.11 and p = 0.051 versus low). No statistically significant survival differences were observed between income groups in those treated with systemic therapy (Fig. 3 & Supplementary Table S7).

### Oesophageal cancer treatment and survival related to SES

In 10,224 potentially curable oesophageal AC patients, neoadjuvant chemoradiotherapy followed by surgery was performed in 30.1% of low-income patients, 39.9% of middle-income patients, and 43.5% of high-income patients (p < 0.001). Best supportive care was most frequently chosen in the low-income group (29.8%) compared to the middle-income (19.9%) and high-income groups (15.7%) (p < 0.001) (Table 2).

For palliative oesophageal adenocarcinoma patients, systemic therapy was administered in 35.2% of low-income patients, 45.4% of middle-income patients, and 51.5% of high-income patients (p < 0.001). Best supportive care was more common in the low-income group (60.3%) compared to the middle-income (49.5%) and high-income groups (43.0%) (p < 0.001) (Table 2).

The 3739 patients with potentially curable SCC receiving neoadjuvant chemoradiotherapy with resection ± nivolumab was 17.3% in low-income patients, 22.2% in middle-income patients, and 26.4% in high-income patients (p < 0.001). The patients with potentially curable SCC patients receiving definitive chemoradiotherapy was 26.6% in the low-income group, 31.3% in the high-income group, and 27.5% in the high-income group. For both curable and palliative SCC and AC patients, the proportion receiving only best supportive care was greatest in the low-income group (Tables 2 and 4).

Multivariable logistic regression showed increased odds of proceeding to surgery for middle-income versus low-income patients (OR 1.17, 95% CI: 1.57–1.30, p < 0.001) and high-income versus low-income patients (OR 1.27, 95% CI: 1.13–1.43, p < 0.001) (Table 3). Similarly, in the palliative setting, multivariable logistic regression showed increased odds of receiving palliative therapy for middle-income versus low-income patients (OR 1.38, 95% CI: 1.21–1.57, p < 0.001) and high-income versus low-income patients (OR 1.53, 95% CI: 1.34–1.74, p < 0.001) (Table 5).

### Relative survival among oesophageal adenocarcinoma patients

Among potentially curable oesophageal AC patients, median relative survival was 23.4 months (95% CI: 21.9–25.0) in the low-income group, 26.0 months (95% CI: 24.1–27.9) in the middle-income group (p < 0.001 versus low), and 30.2 months (95% CI: 27.8–32.6) in the high-income group (p < 0.001 versus low). The 5-year relative survival rate was 28.8% (95% CI: 26.7–30.8), 32.0% (95% CI: 30.1–34.0), and 36.1% (95% CI: 34.1–38.0) for the low-, middle-, and high-income groups, respectively (all p < 0.001 versus low) (Fig. 2 & Supplementary Table S6).

In the subgroup of surgically treated patients, median relative survival was 38.4 months (95% CI: 33.2–43.7) in the low-income group, 42.4 months (95% CI: 36.2–48.5) in the middle-income group (p = 0.46 versus low), and 44.2 months (95% CI: 39.2–49.1) in the high-income group (p = 0.93 versus low). The corresponding 5-year survival rates were 55.9% (95% CI: 47.6–63.4), 59.1% (95% CI: 52.2–65.3), and 52.9% (95% CI: 45.2–60.0), with no statistically significant differences observed (Fig. 2 & Supplementary Table S6).

For patients with metastatic disease, median relative survival was 4.7 months (95% CI: 4.3–5.0) in the low-income group, 5.6 months (95% CI: 5.2–6.0) in the middle-income group (p < 0.001 versus low), and 6.4 months (95% CI: 5.9–6.9) in the high-income group (p < 0.001 versus low). The 1-year survival rate was 21.5% (95% CI: 19.6–23.6), 25.2% (95% CI: 23.4–27.0), and 28.3% (95% CI: 26.3–30.2), respectively (all p < 0.001 versus low). No statistically significant survival differences were observed between income groups in patients treated with systemic therapy (Fig. 3 & Supplementary Table S7).

### Relative survival among oesophageal squamous cell carcinoma patients

In potentially curable ESCC patients, median relative survival was significantly longer in the middle- and high-income groups compared to the low-income group (23.6 and 25.3 versus 18.7 months; both p < 0.001). Similarly, 5-year survival rates were higher in the middle- (32.7%) and high-income groups (34.8%) than in the low-income group (28.5%; both p < 0.001) (Fig. 2 & Supplementary Table S6).

Among surgically treated patients, the 5-year survival rates were 55.9%, 59.1%, and 52.9% in the low-, middle-, and high-income groups, respectively, with no statistically significant differences (p = 0.46 and p = 0.93 versus low) (Fig. 2 & Supplementary Table S6). In patients with metastatic disease, median survival increased with income (4.5 versus 5.4 versus 7.3 months; p = 0.06 and p < 0.001 versus low), as did 1-year survival (20.1%, 21.9%, and 31.3%; p = 0.06 and p < 0.001 versus low). No significant survival differences were found between income groups in those receiving systemic therapy (Fig. 3 & Supplementary Table S7).

## Discussion

This nationwide, population-based study of 30,184 gastroesophageal cancer patients identified significant socioeconomic status-based disparities in cancer care in the Netherlands. These disparities were evident in patients' characteristics, treatment allocation, and survival outcomes. In both gastric and oesophageal cancer, high-income patients were more likely to receive curative treatment or systemic therapy, while low-income patients more often received only supportive care, with disparities being most evident in the palliative setting. These findings suggest that socioeconomic barriers, despite a healthcare system designed for equal access, continue to affect patients’ ability to receive timely or optimal care.

The observed disparities are unlikely to result from differences in formal access to care. In the Netherlands, all residents have mandatory health insurance covering surgical and oncologic treatment nationwide. Lower treatment rates among low-income patients therefore likely reflect patient- and provider-level factors, such as comorbidity, fitness, perceived treatment burden, or communication barriers, rather than financial or institutional restrictions. Equal coverage does not necessarily ensure equal utilization of complex multimodality care.

Comorbidities were more common and performance status was lower in low-income patients, possibly related to health behaviours and educational level, which could reduce eligibility for surgical resection or systemic therapy and increase the likelihood of receiving only best supportive care.^26^^,^^27^ Chronic disease may also lower income by limiting employment and financial stability.^28^

Overall patient fitness is a crucial determinant of treatment eligibility, as multiple chronic conditions reduce physiological reserves and limit suitability for intensive therapies, while lower baseline fitness, independent of comorbidities, may also contribute to disparities, with low-income patients often having reduced physical resilience.^29^ Lifestyle-related risk factors such as smoking, alcohol use, poor diet, and physical inactivity are more prevalent in low-income groups and increase both comorbidity burden and treatment intolerance. Limited access to preventive healthcare services may further impair the management of chronic conditions, leading to poorer health status at diagnosis.^30^ Finally, economic constraints can negatively affect diet and nutrition, contributing to frailty and sarcopenia, which in turn lower the likelihood of eligibility for multimodality treatment.^31^ Hence, the observed inequalities likely reflect not only differences in care delivery but also broader social and biological determinants established before cancer diagnosis.

Importantly, adjustment for comorbidity and performance status did not remove the observed disparities in treatment allocation. High-income oesophageal cancer patients remained more likely to undergo surgery than low-income patients, and middle-income gastric cancer patients more likely than those with low income. In line with earlier research, the lowest income group contained a higher proportion of females (37.9% versus 27.6% in the highest group),^32^ suggesting a gender imbalance, although SES classification by household income does not fully capture individual gender composition.

Relative survival was consistently lower in low-income patients, and previous research suggests that lower adherence to guideline-concordant treatment may contribute to these disparities.^33^, ^34^, ^35^, ^36^ Overall, these findings indicate that SES-related differences in outcomes are not fully explained by measurable clinical factors but reflect broader socioeconomic and systemic influences.

Unexpectedly, metastatic disease was more common in higher-income patients, in contrast to previous reports of later-stage diagnoses in lower-income groups.^37^^,^^38^ Our study cannot determine overall differences in stage distribution, as analyses were restricted to stage-specific subgroups, but these findings suggest that SES-related survival disparities are not solely explained by stage at diagnosis. Instead, they may reflect systemic or financial barriers to multimodality care, or differences in staging intensity.^39^ Higher-income patients, who typically have better performance status and fewer comorbidities, are more often selected for curative-intent strategies and therefore undergo extensive staging such as PET-CT or diagnostic laparoscopy, which may reveal occult metastases that remain undetected in other groups.^40^ Differences in healthcare-seeking behaviour, symptom interpretation, and referral pathways could also contribute, with higher-income patients potentially accessing specialist services and comprehensive diagnostic protocols more rapidly than those with lower income.^41^^,^^42^ This differential staging intensity may have important implications for stage-specific survival comparisons. Less extensive staging in lower-income patients may lead to under detection of metastatic disease and subsequent stage migration, potentially resulting in an overestimation of true survival differences between socioeconomic groups within the same stage.

A key strength of this study is that it is the first population-based analysis to incorporate socioeconomic status in such a large cohort of patients with gastroesophageal cancer. Another strength is the reliance on the prospectively collected NCR, which systematically records staging and treatment data, enabling comprehensive evaluation of both treated and untreated patients. Limitations include the absence of information on treatment decision-making, such as physicians’ initial plans and reasons for deviating from guidelines. Therefore, it is not possible to determine whether the lower treatment rates in low-income groups reflect clinical ineligibility, patient choice, or provider-related factors. Furthermore, the registry does not allow distinction between intentional definitive chemoradiotherapy and planned neoadjuvant chemoradiotherapy not followed by surgery. This may have led to some misclassification of treatment intent. However, as this limitation applies equally across SES, systematic bias is unlikely. Although comorbidities were more frequent in low-income patients, adjustment did not eliminate disparities, indicating that additional socioeconomic and system-level determinants contribute. Importantly, education level was not available, this factor is closely linked to healthcare navigation and decision-making and could partially explain the observed disparities. Their absence likely results in an underestimation of the true scope of socioeconomic inequalities. Integration of such variables, as achieved in other national registries such as in Norway, may strengthen future analyses.^43^ Furthermore, the SES measure was area-based and derived from 2019 CBS data, which may not capture income changes over time. In addition, the original twelve CBS-defined income categories were combined into three broader groups to maintain statistical robustness and interpretability, which may have reduced granularity at the higher end of the income spectrum. However, this simplification was necessary to avoid partially overlapping income categories inherent to the CBS classification, which incorporates 99% confidence intervals of median income at postal code level and may otherwise compromise interpretability in multivariable analyses. Although comorbidities were accounted for using the Charlson categories, residual confounding cannot be fully excluded, as the registry lacks information on comorbidity severity or functional impact. Taken together, these limitations underline that socioeconomic disparities in gastroesophageal cancer care likely arise from a complex interplay of patient-level, provider-level, and system-level factors rather than financial barriers alone.

To better understand and reduce SES-related disparities in cancer treatment, further research is needed. Future analyses could additionally incorporate urbanization or regional accessibility measures to explore potential geographic contributors to socioeconomic disparities. Qualitative studies could uncover barriers in patient-provider decision-making. Patient navigation programs and case managers may help address gaps in healthcare-seeking behaviour and system navigation. Training to reduce implicit bias and support shared decision-making could improve equity in clinical recommendations. Public health strategies that promote healthy lifestyle behaviours could be especially important for populations with lower socioeconomic status, who are disproportionately affected by cancer risk.^44^ Finally, routine registration of individual educational level, as practiced in Norway, would improve the precision of future research and policy targeting.^45^

### Conclusion

Socioeconomic disparities in gastroesophageal cancer outcomes were substantial. Low-income patients had lower relative survival and were less likely to receive surgery or systemic therapy compared with middle- and high-income patients. Despite the Netherlands’ mandatory health insurance system, socioeconomic disparities in treatment outcomes persist, suggesting that factors beyond insurance coverage continue to influence care. The observed disparities therefore likely reflect broader life-course health inequalities rather than deficiencies in the cancer-care system itself. This study highlights the necessity of targeted interventions to reduce these inequities and offers valuable insights for advancing healthcare equity on a global scale.

## Contributors

RHAV and MIvB designed the study. DCvdA performed the data and statistical analysis and drafted the manuscript. RHAV, MIvB and MDT directly accessed and verified the underlying data and analysis. MDT contributed to manuscript drafting and revision of the manuscript. HWMvL, SSG, SML, NHM, and PSNvR contributed to study conceptualization, interpretation of results, and critical revision of the manuscript. RHAV coordinated data acquisition from the Netherlands Cancer Registry, contributed to study design and data interpretation, and critically revised the manuscript. MIvB provided overall supervision, contributed to study design and interpretation, and critically revised the manuscript. All authors had full access to the data, contributed to interpretation of findings, and approved the final manuscript. RHAV and MIvB share senior authorship.

## Data sharing statement

Following publication, data may be shared with qualified researchers upon reasonable request to the corresponding author. Detailed pseudonymised individual-level data cannot be shared due to the risk of potential re-identification.

## Declaration of interests

Rob Verhoeven has received research grants from Bristol Myers Squibb and Amgen and performed consultancy for Daiichi Sankyo, all paid to the institute. Hanneke W. M. van Laarhoven received Research funding and/or medication supply: Amphera, Anocca, Astellas, AstraZeneca, Beigene, Boehringer, Daiichy-Sankyo, Dragonfly, MSD, Myeloid, ORCA, Servier. Consultant/advisory role: Auristone, Incyte, Merck, Myeloid, Servier. Speaker role: Astellas, AstraZeneca, Beigene, Benecke, BMS, Daiichy-Sankyo, JAAP, Medtalks, Novartis, Springer, Travel Congress Management B.V. Nadia Haj Mohammad received consulting fees from Astra Zeneca, BMS, Merck, Servier and research funding from Servier, funding for speaker role from Astra Zeneca, Jaap, Medtalks. Suzanne S. Gisbertz is consultant for Medicaroid, J&J and Olympus. Mark I. van Berge Henegouwen serves in a consulting or advisory role for Alesi Surgical, BBraun, Johnson and Johnson, Medtronic, and Viatris and receives research grants from Stryker outside the submitted work. The remaining authors report no conflicts of interest.
