# Supplementary material for: Socioeconomic disparities in treatment and survival of oesophageal and gastric cancer in the Netherlands: a nationwide population-based study
Source: Lancet Reg Health Eur. 2026 Mar 26;65:101662. doi: 10.1016/j.lanepe.2026.101662 (PMC13050078; doi:10.1016/j.lanepe.2026.101662)
Supplement: Supplementary Tables [file mmc1.docx]

**SUPPLEMENTARY TABLES**

**Table 6.** Relative median and five-year survival in potentially curable gastroesophageal cancer patients

| **Cancer Type** | **Subgroup** | **Low-income** | **Middle-income** | **High-income** | **Low-income** | **Middle-income** | **High-income** | **p-value** |
| --- | --- | --- | --- | --- | --- | --- | --- | --- |
|  |  | **Median**  **(95% CI)** | **Median**  **(95% CI)** | **Median**  **(95% CI)** | **5-yr (%)**  **(95% CI)** | **5-yr (%)**  **(95% CI)** | **5-yr (%)**  **(95% CI)** |  |
| GAC | All patients | 28.5 (24.4–32.4) | 35.6 (32.4–38.8) | 34.4 (28.5–40.1) | 36.5 (33.6–39.4) | 41.1 (38.2–44.0) | 39.3 (35.8–42.8) | <0.001 |
| GAC | Surgery | 51.1 (43.2–59.0) | 54.2 (37.6–70.8) | 57.6 (42.3–73.1) | 46.0 (42.4–49.4) | 48.1 (44.7–51.4) | 49.7 (45.5–53.8) | 0.010 |
| EAC | All patients | 23.4 (21.9–25.0) | 26.0 (24.1–27.9) | 30.2 (27.8–32.6) | 28.8 (26.7–30.8) | 32.0 (30.1–34.0) | 36.1 (34.1–38.0) | <0.001 |
| EAC | Surgery | 38.4 (33.2–43.7) | 42.4 (36.2–48.5) | 44.2 (39.2–49.1) | 55.9 (47.6–63.4) | 59.1 (52.2–65.3) | 52.9 (45.2–60.0) | 0.930 |
| ESCC | All patients | 18.7 (16.7–20.8) | 23.6 (19.4–27.8) | 25.3 (21.5–29.0) | 28.5 (24.7–32.5) | 32.7 (29.2–36.2) | 34.8 (30.4–39.3) | <0.001 |
| Abbreviations: CI, confidence interval; GAC, gastric adenocarcinoma (n=4,158); EAC, esophageal adenocarcinoma (n=10,224); ESSC, esophageal squamous cell carcinoma (n=3,739); yr, year | | | | | | | | |

**Table 7.** Relative median and one-year survival in palliative gastroesophageal cancer patients

| **Cancer Type** | **Subgroup** | **Low-income** | **Middle-income** | **High-income** | **Low-income** | **Middle-income** | **High-income** | **p-value** |
| --- | --- | --- | --- | --- | --- | --- | --- | --- |
|  |  | **Median (95% CI)** | **Median (95% CI)** | **Median**  **(95% CI)** | **1-yr (%)**  **(95% CI)** | **1-yr (%)**  **(95% CI)** | **1-yr (%)**  **(95% CI)** |  |
| GAC | All M1 patients | 4.2 (3.9–4.6) | 4.9 (4.4–5.3) | 4.8 (4.1–5.5) | 19.5 (17.4–21.7) | 21.1 (18.9–23.5) | 22.6 (20.0–25.4) | 0.051 |
| EAC | All M1 patients | 4.7 (4.3–5.0) | 5.6 (5.2–6.0) | 6.4 (5.9–6.9) | 21.5 (19.6–23.6) | 25.2 (23.4–27.0) | 28.3 (26.3–30.2) | <0.001 |
| ESCC | All M1 patients | 4.5 (3.9–5.2) | 5.4 (4.5–6.4) | 7.3 (6.2–8.4) | 20.1 (16.4–24.1) | 21.9 (18.7–25.3) | 31.3 (26.4–36.2) | <0.001 |
| GAC | Systemic therapy | 8.2 (7.4–9.0) | 9.1 (8.4–9.8) | 9.3 (8.6–10.0) | 34.2 (29.9–38.6) | 33.8 (29.7–38.0) | 33.9 (29.3–38.6) | 0.200 |
| EAC | Systemic therapy | 9.6 (9.0–10.2) | 9.5 (8.9–9.9) | 10.0 (9.3–10.6) | 39.0 (35.1–43.0) | 40.2 (36.9–43.5) | 40.8 (37.5–44.1) | 0.430 |
| ESCC | Systemic therapy | 7.0 (5.8–8.3) | 8.0 (6.0–9.8) | 8.9 (6.3–11.3) | 30.3 (19.0–42.4) | 29.1 (21.5–37.1) | 33.7 (23.7–43.9) | 0.300 |
| Abbreviations: CI, confidence interval; GAC, gastric adenocarcinoma (n=3,804); EAC, esophageal adenocarcinoma (n=6,673); ESSC, esophageal adenocarcinoma (n=6,673); yr, year | | | | | | | | |

**Table 8.** Analysis of proceeding to surgery in potentially curable esophageal cancer patients

| **Esophageal cancer** | | | | |
| --- | --- | --- | --- | --- |
|  | **Univariable** | | **Multivariable** | |
|  | **OR (95% CI)** | **p-value** | **OR (95% CI)** | **p-value** |
| **Socioeconomic status** |  |  |  |  |
| **Low-income** | ref |  | ref |  |
| **Middle-income** | 1.54 (1.41-1.68) | <0.001 | 1.17 (1.05-1.30) | 0.005 |
| **High-income** | 1.99 (1.83-2.18) | <0.001 | 1.27 (1.13-1.43) | <0.001 |
| **Gender** |  |  |  |  |
| **Female** | 0.58 (0.54-0.63) | <0.001 | 0.94 (0.85-1.05) | 0.275 |
| **Age** |  |  |  |  |
| **<50 years** | ref |  | ref |  |
| **50-59 years** | 0.56 (0.43-0.74) | <0.001 | 0.62 (0.45-0.85) | 0.003 |
| **60-69 years** | 0.39 (0.30 -0.51) | <0.001 | 0.52 (0.39 -0.71) | <0.001 |
| **70-79 years** | 0.19 (0.14-0.24) | <0.001 | 0.29 (0.21-0.39) | <0.001 |
| **80> years** | 0.02 (0.01-0.02) | <0.001 | 0.03 (0.02-0.04) | <0.001 |
| **Tumor histology** |  |  |  |  |
| **Squamous cell carcinoma** | 0.37 (0.34-0.40) | <0.001 | 0.29 (0.26-0.32) | <0.001 |
| **Clinical Tumor Stage (cT)** |  |  |  |  |
| **1** | ref |  | ref |  |
| **2** | 0.58 (0.45-0.74) | <0.001 | 0.45 (0.33-0.62) | <0.001 |
| **3** | 0.67 (0.53-0.86) | <0.001 | 0.52 (0.38-0.71) | <0.001 |
| **4** | 0.35 (0.25-0.50) | <0.001 | 0.29 (0.19-0.45) | <0.001 |
| **Unknown** | 0.12 (0.09-0.16) | <0.001 | 0.23 (0.16-0.34) | <0.001 |
| **Clinical Nodal Stage (cN)** |  |  |  |  |
| **0** | ref |  | ref |  |
| **1** | 1.01 (0.93-1.10) | 0.763 | 0.84 (0.75-0.93) | <0.001 |
| **2** | 1.03 (0.93-1.14) | 0.529 | 0.73 (0.64-0.83) | <0.001 |
| **3** | 0.78 (0.62-0.97) | 0.027 | 0.44 (0.34-0.59) | <0.001 |
| **Unknown** | 0.09 (0.06-0.11) | <0.001 | 0.24 (0.17-0.35) | <0.001 |
| **Therapy regions** |  |  |  |  |
| **1** | ref |  | ref |  |
| **2** | 0.45 (0.38-0.53) | <0.001 | 0.33 (0.27-0.41) | <0.001 |
| **3** | 0.68 (0.59-0.80) | <0.001 | 0.59 (0.48-0.73) | <0.001 |
| **4** | 0.76 (0.65-0.89) | <0.001 | 0.61 (0.50-0.75) | <0.001 |
| **5** | 0.65 (0.54-0.77) | <0.001 | 0.59 (0.47-0.75) | <0.001 |
| **6** | 0.59 (0.50-0.70) | <0.001 | 0.41 (0.33-0.51) | <0.001 |
| **7** | 0.57 (0.49-0.67) | <0.001 | 0.50 (0.40-0.60) | <0.001 |
| **WHO performance score** |  |  |  |  |
| **0** | ref |  | ref |  |
| **1** | 0.39 (0.36-0.43) | <0.001 | 0.51 (0.46-0.56) | <0.001 |
| **≥2** | 0.05 (0.04-0.06) | <0.001 | 0.09 (0.08-0.11) | <0.001 |
| **Unknown** | 0.16 (0.15-0.18) | <0.001 | 0.35 (0.30-0.40) | <0.001 |
| **Number of CCI categories** |  |  |  |  |
| **0** | ref |  | ref |  |
| **1** | 0.57 (0.52-0.62) | <0.001 | 0.77 (0.69-0.85) | <0.001 |
| **≥2** | 0.27 (0.24-0.30) | <0.001 | 0.48 (0.43-0.55) | <0.001 |
| Abbreviations: OR, Odds Ratio; CI, Confidence Interval; CCI: comorbidity categories index; WHO: World Health Organization | | | | |

**Table 9.** Analysis of proceeding to systemic therapy in palliative esophageal cancer patients

| **Esophageal cancer** | | | | |
| --- | --- | --- | --- | --- |
|  | **Univariable** | | **Multivariable** | |
|  | **OR (95% CI)** | **p-value** | **OR (95% CI)** | **p-value** |
| **Socioeconomic status** |  |  |  |  |
| **Low-income** | ref |  | ref |  |
| **Middle-income** | 1.54 (1.38-1.72) | <0.001 | 1.38 (1.21-1.57) | <0.001 |
| **High-income** | 1.88 (1.68-2.10) | <0.001 | 1.53 (1.34-1.74) | <0.001 |
| **Gender** |  |  |  |  |
| **Female** | 0.64 (0.57-0.70) | <0.001 | 0.79 (0.70-0.89) | <0.001 |
| **Age** | 0.94 (0.93-0.94) | <0.001 | 0.95 (0.94-0.95) | <0.001 |
| **Tumor histology** |  |  |  |  |
| **Squamous cell carcinoma** | 0.29 (0.26-0.33) | <0.001 | 0.30(0.26-0.34) | <0.001 |
| **Therapy regions** |  |  |  |  |
| **1** | ref |  | ref |  |
| **2** | 1.00 (0.83-1.20) | 0.984 | 0.96 (0.77-1.19) | 0.697 |
| **3** | 1.54 (1.29-1.84) | <0.001 | 1.61 (1.31-1.97) | <0.001 |
| **4** | 1.22 (1.01-1.46) | 0.035 | 1.31 (1.06-1.61) | 0.012 |
| **5** | 1.04 (0.85-1.28) | 0.704 | 1.23 (0.96-1.57) | 0.100 |
| **6** | 1.23 (1.00-1.50) | 0.044 | 1.15 (0.91-1.45) | 0.234 |
| **7** | 1.38 (1.16-1.64) | <0.001 | 1.54 (1.26-1.88) | <0.001 |
| **WHO performance score** |  |  |  |  |
| **0** | ref |  | ref |  |
| **1** | 0.72 (0.64-0.82) | <0.001 | 0.21 (0.18-0.25) | 0.008 |
| **≥2** | 0.17 (0.15-0.20) | <0.001 | 0.28 (0.24-0.33) | <0.001 |
| **Unknown** | 0.24 (0.21-0.28) | <0.001 | 0.26 (0.23-0.30) | <0.001 |
| **Number of CCI categories** |  |  |  |  |
| **0** | ref |  | ref |  |
| **1** | 0.69 (0.62-0.76) | <0.001 | 0.85 (0.76-0.95) | 0.005 |
| **≥2** | 0.43 (0.38-0.49) | <0.001 | 0.64(0.55-0.74) | <0.001 |
| Abbreviations: OR, Odds Ratio; CI, Confidence Interval; CCI: comorbidity categories index; WHO: World Health Organization | | | | |

| **Gastric cancer** | | | | |
| --- | --- | --- | --- | --- |
|  | **Univariable** | | **Multivariable** | |
|  | **OR (95% CI)** | **p-value** | **OR (95% CI)** | **p-value** |
| **Socioeconomic status** |  |  |  |  |
| **Low-income** | ref |  | ref |  |
| **Middle-income** | 1.72 (1.48-2.00) | <0.001 | 1.48 (1.21-1.80) | <0.001 |
| **High-income** | 1.45 (1.23-1.72) | <0.001 | 1.02 (0.82-1.27) | 0.854 |
| **Gender** |  |  |  |  |
| **Female** | 0.93 (0.82-1.06) | 0.297 | 1.11 (0.93–1.33) | 0.241 |
| **Age** |  |  |  |  |
| **<50 years** | ref |  | ref |  |
| **50-59 years** | 0.79 (0.45–1.41) | 0.431 | 0.59 (0.28–1.21) | 0.148 |
| **60-69 years** | 0.49 (0.30–0.82) | 0.006 | 0.39 (0.20–0.75) | 0.005 |
| **70-79 years** | 0.29 (0.18–0.46) | <0.001 | 0.26 (0.14–0.50) | <0.001 |
| **80> years** | 0.05 (0.03–0.09) | <0.001 | 0.06 (0.03–0.12) | <0.001 |
| **Lauren classification** |  |  |  |  |
| **Intestinal** | ref |  | ref |  |
| **Diffuse** | 0.74 (0.64-0.86) | <0.001 | 0.48 (0.40–0.59) | <0.001 |
| **Mixed** | 1.02 (0.72-1.45) | 0.909 | 0.77 (0.50–1.18) | 0.228 |
| **Unknown** | 0.36 (0.29-0.44) | <0.001 | 0.32 (0.24–0.42) | <0.001 |
| **Clinical Tumor Stage (cT)** |  |  |  |  |
| **1** | ref |  | ref |  |
| **2** | 0.69 (0.44-1,10) | 0.121 | 0.67 (0.36–1.24) | 0.198 |
| **3** | 0.52 (0.33-0.81) | <0.001 | 0.51 (0.28–0.95) | 0.034 |
| **4** | 0.47 (0.28-0.79) | <0.001 | 0.45 (0.22–0.89) | 0.023 |
| **Unknown** | 0.17 (0.11-0.27) | <0.001 | 0.30 (0.16–0.56) | <0.001 |
| **Clinical Nodal Stage (cN)** |  |  |  |  |
| **0** | ref |  | ref |  |
| **1** | 0.89 (0.75-1.07) | 0.213 | 0.68 (0.54–0.84) | <0.001 |
| **2** | 0.63 (0.50-0.79) | <0.001 | 0.46 (0.34–0.62) | <0.001 |
| **3** | 0.89 (0.07-0.12) | <0.001 | 0.15 (0.11–0.21) | <0.001 |
| **Therapy regions** |  |  |  |  |
| **1** | ref |  | ref |  |
| **2** | 0.80 (0.59-1.07) | 0.127 | 0.56 (0.37–0.85) | 0.006 |
| **3** | 1.05 (0.79-1.38) | 0.756 | 0.76 (0.51–1.13) | 0.172 |
| **4** | 1.34 (1.00-1.79) | 0.047 | 1.11 (0.75–1.66) | 0.600 |
| **5** | 1.09 (0.79-1.51) | 0.604 | 0.90 (0.57–1.42) | 0.649 |
| **6** | 0.96 (0.72-1.29) | 0.795 | 0.71 (0.48–1.07) | 0.100 |
| **7** | 0.93 (0.70-1.21) | 0.574 | 0.92 (0.63–1.35) | 0.681 |
| **WHO performance score** |  |  |  |  |
| **0** | ref |  | ref |  |
| **1** | 0.51 (0.41-0.64) | <0.001 | 0.64 (0.50–0.83) | <0.001 |
| **≥2** | 0.09 (0.07-0.12) | <0.001 | 0.16 (0.12–0.21) | <0.001 |
| **Unknown** | 0.15 (0.12-0.18) | <0.001 | 0.30 (0.23–0.39 | <0.001 |
| **Number of CCI categories** |  |  |  |  |
| **0** | ref |  | ref |  |
| **1** | 0.60 (0.51-0.70) | <0.001 | 0.81 (0.66–1.00) | 0.048 |
| **≥2** | 0.37 (0.31-0.44) | <0.001 | 0.65 (0.52–0.81) | <0.001 |
| Abbreviations: OR, Odds Ratio; CI, Confidence Interval; CCI: comorbidity categories index; WHO: World Health Organization | | | | |

**Table 10.** Analysis of proceeding to surgery in potentially curable gastric cancer patients

**Table 11.** Analysis of proceeding to systemic therapy in palliative gastric cancer patients

| **Gastric cancer** | | | | |
| --- | --- | --- | --- | --- |
|  | **Univariable** | | **Multivariable** | |
|  | **OR (95% CI)** | **p-value** | **OR (95% CI)** | **p-value** |
| **Socioeconomic status** |  |  |  |  |
| **Low-income** | ref |  | ref |  |
| **Middle-income** | 1.36 (1.17-1.59) | <0.001 | 1.20 (0.99-1.44) | 0.050 |
| **High-income** | 1.49 (1.26-1.76) | <0.001 | 1.27 (1.05-1.55) | 0.014 |
| **Gender** |  |  |  |  |
| **Female** | 0.94 (0.82-1.08) | 0.370 | 0.90 (0.76-1.05) | 0.174 |
| **Age** | 0.94 (0.93-0.95) | <0.001 | 0.94 (0.93-0.95) | <0.001 |
| **Lauren classification** |  |  |  |  |
| **Intestinal** | ref |  | ref |  |
| **Diffuse** | 1.16 (1.00-1.35) | 0.056 | 0.84 (0.70-1.00) | 0.051 |
| **Mixed** | 1.07 (0.73-1.54) | 0.753 | 0.70 (0.45-1.08) | 0.108 |
| **Unknown** | 0.86 (0.71-1.06) | 0.153 | 0.87 (0.69-1.10) | 0.236 |
| **Therapy regions** |  |  |  |  |
| **1** | ref |  | ref |  |
| **2** | 0.77 (0.57-1.05) | 0.098 | 0.76 (0.53-1.10) | 0.143 |
| **3** | 1.67 (0.96-1.67) | 0.094 | 1.18 (0.85-1.64) | 0.330 |
| **4** | 0.94 (0.70-1.25) | 0.662 | 0.98(0.70-1.38) | 0.907 |
| **5** | 0.81 (0.58-1.14) | 0.236 | 1.02 (0.68-1.53) | 0.925 |
| **6** | 0.88 (0.65-1.18) | 0.383 | 0.91 (0.64-1.29) | 0.594 |
| **7** | 0.77 (0.58-1.02) | 0.066 | 0.76 (0.54-1.05) | 0.096 |
| **WHO performance score** |  |  |  |  |
| **0** | ref |  | ref |  |
| **1** | 0.82 (0.67-0.99) | 0.046 | 0.89 (0.72-1.10) | 0.290 |
| **≥2** | 0.22 (0.18-0.28) | <0.001 | 0.26 (0.20-0.33) | <0.001 |
| **Unknown** | 0.19 (0.15-0.23) | <0.001 | 0.22 (0.17-0.27) | <0.001 |
| **Number of CCI categories** |  |  |  |  |
| **0** | ref |  | ref |  |
| **1** | 0.67 (0.58-0.79) | <0.001 | 0.97 (0.82-1.26) | 0.765 |
| **≥2** | 0.38 (0.31-0.46) | <0.001 | 0.75 (0.60-0.95) | 0.015 |
| Abbreviations: OR, Odds Ratio; CI, Confidence Interval; CCI: comorbidity categories index; WHO: World Health Organization | | | | |
